# COVID-19 policies and the arising of debate on twitter

**DOI:** 10.3389/fsoc.2022.1106393

**Published:** 2023-01-06

**Authors:** Simona Gozzo, Rosario D'Agata

**Affiliations:** Department of Political and Social Sciences, University of Catania, Catania, Italy

**Keywords:** automatic content analysis, social network, network analysis, pandemic, narratives

## Abstract

This study focuses on the analysis of contacts and communications on Twitter concerning pandemic policies. The goal is both show mobilization processes rising from the web and detect main actors, themes, and contents within the European context. Through a mixed method procedure, we tried to identify the main themes and most relevant communities, the main users, the most relevant topics and languages, and the underlined meanings and differences related to languages (as *proxies* of areas). Monitoring the communication on 3 main topics (“no-mask”, “covid-19”, “greenpass”), we noticed the weight of the gap between the government's attempts to communicate information motivating measures geared toward managing the crisis and the perceptions of private users. These perceptions spread through the web with such force the more the emotional, ironic, or polemical plane weighs. In this sense, online communication could be considered a tool for understanding the weight of the interaction between the institutional, social, and private dimensions, with effects on the social construction of identities. Digital communication is becoming an element of this process. The paper describes the “reassuring” role played by the digital community in the construction of ontological forms of security resulting from the construction of a shared digital culture. Results show the emergence of digital communities, structured on reference hubs and standing out from the detected phenomenon, prevalence of idioms and even language structures. The relevant role of the emotional (French), ironic (Italian), protest (English) component is confirmed, but also the changing and *fluid* structure of the debate and the co-presence of many parallel discussion communities.

## 1. Introduction

This study focuses on the analysis of contacts and communications on twitter about pandemic policies. The goal is both showing mobilization processes rising from the web and detecting main actors, themes and contents within the European context. The choice of twitter as a social network to monitor derives from several considerations:

- the political nature of the debate leads us to believe that this social network is particularly useful;
- the widespread and transnational use of the same social network allows you to compare information and narratives in different contexts, using the language as a proxy for the area;
- the possibility of identifying short phrases, videos and links allows you to reconstruct the political and identity profiles.

The main question to be answered is if the online communication about the pandemic phenomenon produced something as social and/or political common feelings. With this in mind, the work monitored the on line communication during different phases of pandemic crisis, identifying a keyword for each. We selected, in particular, three main topics/phases (on an international perspective), named as: “No-mask,” “COVID-19,” “Greenpass.” A specific focus was on the protest communication. When the No-mask protests decreased, the communication centered on the “COVID-19” lemma was monitored. This made it possible to compare the different communication structures, the relative narratives, the weight of (residual) protest communication, and the persistence of dialogue on the more general topic of the pandemic. The “No-mask” and “COVID-19” communication—although centered on the same issue (the effect of the pandemic crisis in social life) differ in structure, persistence, narratives and also characterizing languages. Another communication structure is referred on a specific form of protest: that against the “COVID-pass.” This, compared to the No-mask one, presents some specific characteristics: the greater endurance over time and the particular presence of Italian protests. The decision to make mandatory a document certifying the immunization to allow the free movement of the population, in fact, assumed a different weight in each State and, consequently, the communication on this specific issue is mainly referred to areas in which the weight of the legislation was particularly stringent.

We did not want to analyse comments centered on only one key-word, but to choice the characterizing topic each specific period. This was possible by monitoring both the discussion in the media (newspapers, television programs, etc.) and the trend of the debate on social networks. We opted for an analysis conveyed by the same trend of public opinion, being the risk communication an important part of the social perception and construction of collective meanings (Ansell et al., 2010). The work therefore analyses the effects of the perception of the COVID-19 pandemic and related political interventions, consciously with respect to its construction as a social event.

In fact, can the on line communication be defined as a unique process? The answer is: no, it cannot! First of all, the COVID-19 emergence issue, as social and political topic, changed in time for political relevance, social perception, narratives, as real health threat and consequently for its economic impact. So we distinguish at least three different phases of the pandemic crisis, depending on the social perception, political choices, juridical decisions and subsequent economic impacts (all in a European perspective). These phases are well-identified even through a monitoring of the specific on line communication on twitter.

Twitter as social network is particularly suitable for the purpose because each user has a small number of characters for each message, which can be commented on or shared by others, and at the same time the content and users are usually politically oriented. Moreover, the on line communication was particularly spread between 2020 and 2022, when pandemic crisis affected all European States, limiting direct contacts and encouraging digitization (also as a forced choice). Besides, twitter is a very used platform in all States of European union and in England.

The use and platform availability is very important because comments on social networks (as the off line ones) underline different opinions and communities, interests and intentions and we are interested in evaluating the incidence of choices and opinions but also of the structure of communication from a supranational point of view. At the same times, there are many different questions, opinions, problems related to the COVID-19 crisis.

We tried to identify different facets, as:

- the main themes and most relevant communities;
- the main hubs (if there are any), that is the main users, subjects or entities (associations, organizations, newspapers, etc.) that send and receive a large amount of information;
- the most relevant topics and languages, even in a comparative perspective;
- the underlined meanings and differences related to languages (as *proxis* of areas).

During some phase and period, issues have certainly produced more than just a virtual protests or reactions in many European States. On the other hand, we also note some local specificities both with respect to themes and issues, and with respect to the narratives characterizing the area. It is interesting to note, for example, how in the early stages, shortly after the tragedy that hit Italy (the first European area to face the pandemic in particularly problematic conditions) many of the Italian comments extracted on the “No-mask” keyword, are aggressive or ironic, but however opposed to the movement.

## 2. Materials and methods

While certainly one of the key actions of the government during the pandemic crisis was to “make sense” of the crisis, conveying meanings and informing about risks and related decisions, the acceptance of this meanings did not always happen.

Most emergency risk communication is based on the assumption that technical and social improvements in the warning system can motivate people to take protective action. Contrary to popular belief, it has become particularly clear how communicating (even high) risks to the population does not automatically lead to changes in behavior (Floyd et al., 2000; Lindell and Perry, 2012). The work presented aims, therefore, to reconstruct the collective narratives related to the pandemic emergency by adopting different points of view and attempting to reconstruct the motivations that led to the prevalence of one or the other position. This type of analysis wants to investigate the reasons of specific behaviors, for or against emergency policies. Certainly an important concept in this sense is that of the social construction of reality (Pellizzoni and Biancheri, 2021), here redefined in terms of digital ethnography (Amaturo and Aragona, 2019) and risk communication, as well as the analysis of social communication to planning social policies (Bamberger, 2016, 2018; Picciotto, 2020).

### 2.1. Ontological insecurity and digital culture

Is virtual experience itself important for the construction of cultural identities? This is an important question for this study. Although this work focuses on the specific analysis of online communication, it must necessarily address this issue as the relationship between the construction of shared political identities and online communication is becoming more and more relevant and certainly it has significantly impacted the topic of reference. In fact, this is an issue that cannot be ignored when confronting the perception and management of policies on the COVID-19 pandemic.

The on line communication could be considered, in this sense, a tool to understand the weight of the interaction between the micro (individual) and macro (social) dimensions referred to the social construction of identities. Digital communication has become such an element of these process because of it is capable to produce meanings, behavioral models and norms.

The communication on social networks today can, in fact, be considered as one of the elements that convey the construction of identity, as it produces interaction (virtual and real) and derives from prior positions and choices. Identity structure, as well as awareness of a reference context or community are all features that help to produce ontological security. This function is particularly important in the period we are dealing with.

Cultural theory (Thompson, 2008; Johnson and Swedlow, 2021) identifies, specifically, four cultural worldviews, which may be relevant for the outcomes on the reconstructed debate: Individualism; Egalitarianism; Hierarchism; Fatalism. According with this perspective, reality is socially constructed through cognitive mechanisms with which people reproduce the expectations of their in-group (even if digitally constructed, as in this case) as a protective reaction against insecurity and fear (…).

This condition can lead to the spread of false information among groups and communities of users, reducing ontological insecurity and relative perception of risk, with results on individual and/or collective behavior (Raffini and Penalva-Verdú, 2022). In this sense, it was useful to monitor in particular communications centered on “No-mask” and on COVID-pass (in particular with respect to the binding use of the Greenpass in Italy). Specifically, the issue of the diffusion of the confirmation bias (Bessi and Quattrociocchi, 2015; Del Vicario et al., 2016) linked to the individual selection of information and news that confirm one's prejudices, including the sources of access to information, is significant, up to the creation of customized user profiles. Besides, other biases may emerge or overlap with this by depending on additional on line or off line relational, social, and cognitive dynamics and/or psychological distress and anxiety (Tei and Fujino, 2022). Furthermore, different studies highlight how behind conflicts there are causes that go beyond disinformation and individualism and that are related to many causes (Bennett and Pfetsch, 2018).

The sharing of misleading communications, controversies and fake news, crossed with a climate of widespread ontological insecurity, usually produces a high risk of distortion in information. Besides, considering the communication on social networks, the algorithmic management of the sending of customized communications intensifies these identification and identity construction mechanisms, initiating forms of access to dis-information that are no longer controlled even by the user himself. The outcome of the process described is the growing polarization of messages and the public on social media. These dynamics, already noted and discussed due to the negative effects on the social, political, and relational level (Del Vicario et al., 2016), have assumed particular relevance for the possible effects on the health level, linked to the diffusion of opinions contrary to vaccination and, more generally, against every precaution aimed at limiting the increase in infections and the most harmful effects of the pandemic.

The debate that emerged on social media and, more generally, on the media, has produced unprecedented fractures, including transversal ones on the political level, which have led—in Italy—to the emergence of positions defined as communitarian, liberal, progressive and radical (Battistelli and Galatino, 2020). The role of ideology for the cognitive management of ontological insecurity is, in this sense, relevant. People with more political knowledge have, in particular, more developed ideological positions (Carpini and Keeter, 1996). Likewise, cultural worldviews are generally more consistent and their influences are stronger among people with political knowledge (Gastil et al., 2011). So, we can distinguish multiple reactions to the pandemic crisis, alternatively attributable to political ideologies and to the four cultural worldviews identified, especially if we consider the original distinction of individualism vs. communitarianism and egalitarianism vs. hierarchism (Johnson and Swedlow, 2021). In fact, we can identify a communitarian right-wing tending to be alarmist and a liberal right-wing oriented, instead, to minimize the gravity of the pandemic, as well as a progressive left-wing asking for closures as measure extraordinary and a radical left-wing criticism against any closure as risk of authoritarian drift.

### 2.2. Three European phases of pandemic crisis

The pandemic crisis has certainly entailed exceptional and emergency conditions. Everywhere it has been necessary to impose more or less severe measures aimed at limiting infections. In terms of ontological security, the choices made, more oriented toward opening than closing, have certainly influenced the climate of opinion, implying a specific balance between fears of contagion and the risk of impoverishment. These positions certainly emerged from the analysis of the tweets, with a certain distinguishable strength also in terms of different languages, conveying some themes rather than others. As we will note, this implies several stages, characteristics, and related perceptions/definitions. It is interesting to note that the three phases identified through the analysis of the comments and progressive findings that emerged from media monitoring, correspond to as many distinct phases considering the spread of infections in the European context and related more or less binding political decisions (Zupi, 2021). The analysis of the communication on social networks allows to obtain, therefore, information relating to contexts and conditions even distant and different. If this is certainly an advantage, it was decided to limit the analysis only to the European context, which has similar traits and in which the various states have moved with a certain homogeneity. If in this context three specific moments are identifiable (corresponding to the changes in the monitored lemma), the conditions—at least in terms of the reconstructed phases—could change by including comments in other languages.

The first wave of the virus implies a phase of high alert in which almost all European states close schools, many businesses and restrict public gatherings at various levels. Many States also involve the military, reduce public services and introduce night curfews. These decisions trigger protests mainly of a liberal and/or individualist orientation (the “No-mask” protest is born), continuing in Europe also during the second wave of the pandemic (since autumn 2020), in particular during Christmas (December). The second period is, however, a period of loosening of restrictions, in which many European governments decide to adopt a less drastic approach than the previous lockdown, with some alignment of approach but with time and regional lags in implementation.

At the beginning of 2021, many EU countries still adopt restrictive measures, but not comparable to the strict closures of a year earlier: Germany and the United Kingdom adopt a partial lockdown; France, Spain and the Netherlands instead adopt a curfew. In this phase, progressively, the No-mask protest diminishes while communication about the need for closures and the severity of the infection, but also about the economic crisis, remains and intensifies. This is the period in which communication “COVID-19” is monitored. At this stage, on the socio-economic front, European governments are committed, although with different margins for maneuver, to providing massive fiscal, financial and economic support to protect businesses, workers, families and vulnerable populations. National and European public funds are reallocated in favor of the priorities dictated by the crisis, supporting healthcare, vulnerable populations and regions particularly affected by the crisis. These facilitations and supports certainly helps to limit protests and various forms of dissent.

Lastly, and precisely in relation to the centrality of the economic crisis, a third phase of thematic communication on the pandemic emerges, which does not occur in all areas with equal intensity and which refers to prohibitions and controls linked to vaccination and/or immunization. It concerns the need to have a document proving the immunization in order to be able to regularly attend public and private services, offices and facilities. On twitter, the protest centered on the lemma “Greenpass” (the document required and obligatory in Italy) emerges with particular force. This is a phase in which socio-economic benefits are becoming increasingly scarce and, consequently, protest and dissent centered on economic issues are increasing.

### 2.3. Three topics of communication for three features of pandemic crisis

The distinguishable forms of protest and/or discussion in Europe refer to phases and forms of evolution of the online debate, related narratives and limits of mobilization. Although different phases can be identified for each context, linked to the combination of the spread of contagion, political decisions and the different balances that each area has maintained with respect to the need to protect public health and economic interests, the work shows we can distinguish three specific moments of discussion. The first phase of the pandemic was characterized by the “No-mask” protest, linked to the closure and distancing decisions that gradually emerged. In this phase, the presence and mobilization of the protest group is especially widespread in certain areas (England and France) and is characterized by a refusal to adhere to forms of restriction of individual freedom.

The work monitors, at this stage, the recursive online communication between subjects critical of government choices against the risks of contagion underlying the pandemic. We used, in particular, web-scraping tools to extract tweets containing reference lemmas of communication with respect to measures to contain the contagion. Communication centered on “No-mask” was monitored from November 2020 to February 2021.

Subsequently, having noticed a clear reduction in reference communication, the communication centered on “COVID-19” was monitored from March until June 2021. Finally, the last monitoring phase concerned the lemma “Greenpass” (also when associated with variants such as COVID-pass). This observation lasted until December 2021. In March 2022, Italy, which prolonged the state of emergency for a particularly long period, also declared its end.

The decision to monitor the use of these words depends on several considerations, most of which have already been described. Referring specifically to the analysis of the data, the word “No-mask” becomes de facto irrelevant in February 2021, while later on, especially in the summer, dissent becomes macroscopic in relation to the ban on openings and aggregation. Between March and June, there is no other clearly semantically connoted lemma, so we opt to monitor—for comparative and evaluative purposes—the communication on “COVID-19.” This extraction-key makes it possible to distinguish between communication structures and themes. The third phase identified through media monitoring and communication content analysis is the one in which dissent toward emergency measures becomes particularly incisive, especially where—as in Italy—these still persist and strongly condition citizens' lives, especially when they decide not to vaccinate. At this point, the protest again becomes visible and associated with specific lemmas, once again connoting itself on a thematic level. If the headword changes, explicitly leading back to the demand for proof of vaccination (Greenpass, COVID-pass, etc.), the protest re-emerges again in association with the keywords of the first phase (no-vax, No-mask, no-vaccines, etc.). The media visibility of the new wave of protest is evident and trans-national, although it is concentrated in certain areas and—in particular—we centered our analysis in the Italian context.

## 3. Results

Technically, we propose a three-step procedure, which is useful for obtaining information on data extracted from social networks using a number of analysis techniques borrowed from the in-depth qualitative analysis, graph theory and automatic content analysis. Concerning the graph theory, users are defined as nodes and the comments, links and images posted and viewed constitute the links (Hansen et al., 2010, 2012). In this way, the hubs of the network (i.e., those nodes on which the entire network structure depends) are identified and all information regarding the relevance of the messages is reconstructed. Their attractiveness, the potential for intermediation, the selective reach of some messages as opposed to the ability to quickly reach many or all users of others, is information that can be detected using the group analysis function and the various network analysis measures referring to centrality *degree, betweenness*, and *closeness* (Junlong and Yu, 2017).

The same procedure has also been successfully used to analyse other forms of communication on social networks and allows for a kind of controlled, non-automated data mining, skimming relevant information from a potentially infinite database of data and communications (Gozzo and D'Agata, 2020, 2021). This approach captures and integrates information on several levels, cross-referencing the preliminary qualitative analysis of the detectable information on the main network comments with the quantitative investigation of the network structure of the communication, aimed at identifying the most significant structural elements.

Specifically, large samples of tweets were extracted systematically covering the emergence period for the most part of European states: from November 2020 to March 2021. Three main keywords were selected from the debate: No-mask (from November 2020 to February 2021), COVID-19 (from March to June 2021), and green/COVID pass (from June 2021 to December 2021). After collecting edges and comments in 13 waves, we observed different structures of the nets representing links among users. Technically, we remark a three steps procedure applied to obtain complementary information about data extracted from social networks:

1) First of all, we carried out an in-depth (and qualitative) study on the content of the most important tweets. For each extraction, the top-10-tweets, i.e., the most frequent comments among those extracted, were selected, also taking into account the weight of views and re-tweets in the network. The result is an in-depth analysis of the main content posted by the network's hubs, that is the users whose comments were most often viewed and commented on in turn. The qualitative study at this stage allowed us to exclude some tweets, as referred to specific or atypical issues, and to select the main languages for each keyword/period.
2) Then we focused on the analysis of relational structures, employing network analysis tools. This quantitative analysis is carried out on all comments extracted (not only the European ones) by applying network analysis. This step made it possible to detect the eventual presence, weight and quantity of actual parallel communities. It is possible to potentially distinguish a stage of birth, formation and decay of communities by considering the number of reciprocal ties, self-loops, unique ties and the presence of cohesive or non-cohesive subgroups. Finding the presence of small groups and fragmentation of communication on several specific themes indicates a lack of cohesion and therefore of community (even virtual), while detecting large groups shows the structuring of discussions around strong arguments and / or aggregation on thematic points. The analysis is unrolled for each extraction, in order to evaluate how (and if) the network's structure changes. Various considerations are possible with respect to the volatility of the relational and participatory structure, including the aggregation and dispersion of a particularly large number of users on specific themes or moments. This step allowed to select—for each extraction—the communication structure and the main groups as subnets obtained by extracting clusters connected to each other, with greater internal homogeneity and external heterogeneity in terms of links.
3) Finally, the semantic analysis is applied. To perform the automatic content analysis, only main European languages are selected. From the comments in the different European languages, those of the most frequently used languages (identified already in a first stage with qualitative analysis) were selected. This last stage shows differences and specificity on topics and discussions both at the level of languages and periods. This kind of analysis allows to detect the main elementary contexts and themes for each language and to compare thematic clusters with respect to the different periods by progressively employing exploratory co-occurrence analysis, extraction of elementary contexts, identification of clusters of main lemmas and factorial analysis for each language and keyword.

### 3.1. The main users

The first step of the analysis aims at highlighting the Tweets emerged from each extraction. This qualitative step is crucial to determine the network's hub as it is from them that users' reaction will branch. A deeper analysis of the main comments will be needed for a correct matching between the language and the related geographical area.

Certainly, the choice of the extraction keys is going to have an impact on both the structure and the content of the communication. In this case extraction keys are hashtags and they are going to be weighted based on their frequency.

In the first extraction the chosen hashtag is “No-mask.” During this period (from 30th November 2020 to 5th February 2021) five draws were carried out. A close monitoring of the communication showed a high share of comments in Italian, English and French. This first period goes through at least three specific moments of tension: the first vaccine distribution, the growing spread of the contagion and the decision of many Governments to close shops, restaurants and attractions in many parts of the world.

During the second period, ranging from 1st March 2021 to 30th June 2021, governments' measures weakened due to a contagion rate reduction. In order to monitor the communication on twitter, the chosen lemma is “COVID-19.” This generic extraction key has a high incidence of comments in Asiatic languages and English. In particular, most of the comments appear in agreement with the need to impose limitations and restrictions.

Last wave of extraction (from 1st July 2021 to 1st December 2021) refers to the communication centered on the hashtag “Greenpass.” This is specifically characterized by a communication in Italian language, tending to contrast anti-COVID measures. The almost exclusive presence of Italian tweets depends on the hashtag selection. #Greenpass, in fact, is chosen Italian name to refer to a digital COVID-19 certificate, named otherwise all over Europe (France—*passe sanitaire*, Denmark—*coronapass*, etc…) when not specifically named at all.

**Figure 1:**
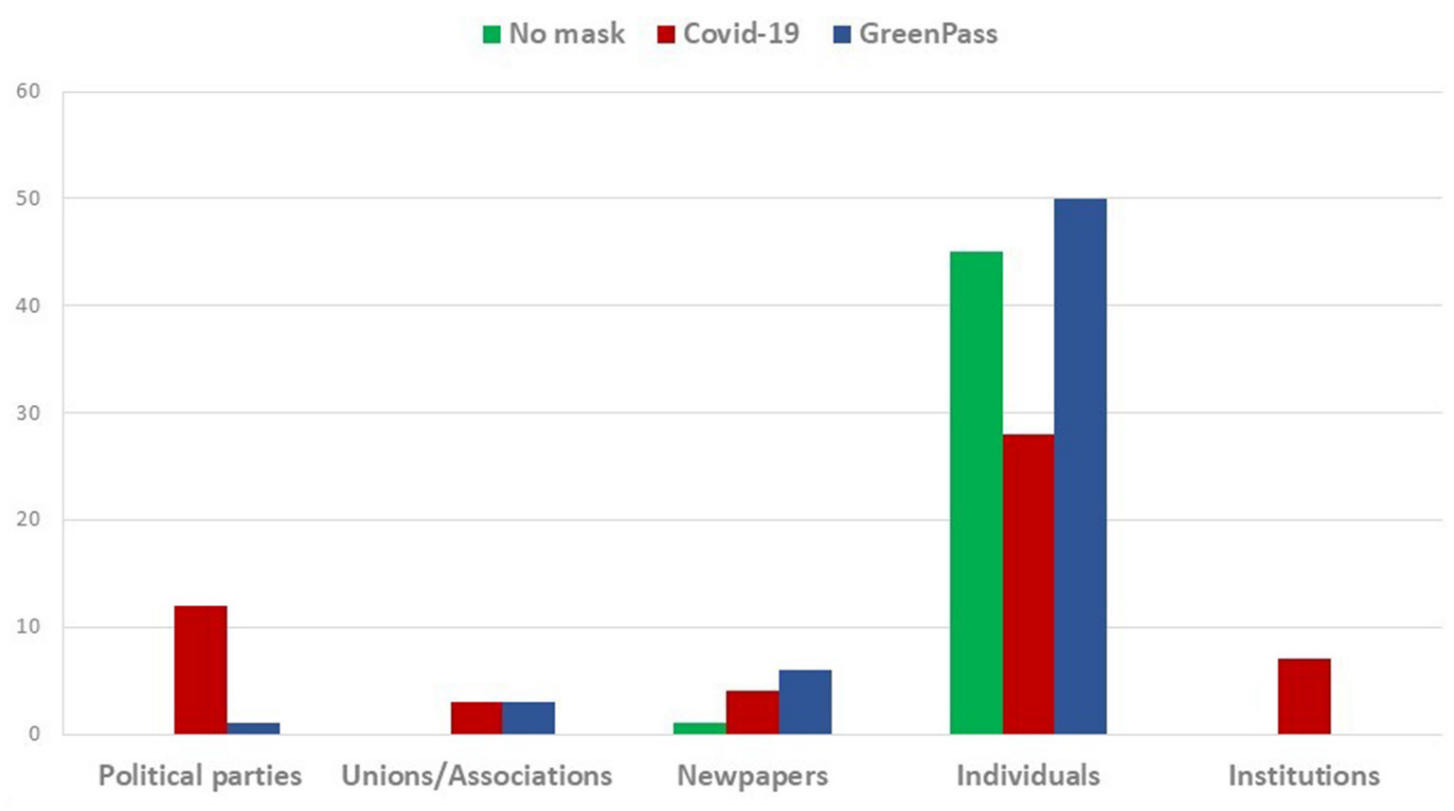
Top 10 tweets: distribution of hashtag for different sources.

This preparatory stage of analysis, as mentioned before, shed light not only on comments and prevalent sentiments but it also gave other relevant information about users, recipients and specific topics. In particular, “No-mask” communication seems to be almost exclusively private and contest-oriented (Figure 1). In fact, while it denotes a satirical approach against no-vax in Italy, it appears more centered on mobility protests and claims to freely purchase in English. If British protests on twitter appear characterized by a form of liberal-populism, in France they seem to be the outcome of a more emotional-individualistic approach; suffice to think about the number of retweets a comment against mask usage in kindergartens had.

As was to be expected, the comments drawn searching the lemma “COVID-19,” are more generic and cover different topics. It emerges, however, a more positive reaction to controls and restrictions. Moreover, this wave shows a marginal importance of no-vax and No-mask claims and—unlike the former wave- comments are posted not only by individuals but also by Institutions like political parties, trade unions, associations and newspapers too.

Hence, it is possible to witness both institutional and non-institutional comments, equally addressed to political institutions and people. The first mainly express dissent to shops' openings against scientific advice, social distancing and mandatory mask usage abolitions. In other cases, these comments tend to criticize the virus mismanagement in schools and prisons. The second, instead, tend either to promote compliance with containment measures (masks wearing, vaccinations, etc.), or contain general updates on the number of infections, the availability of oxygen cylinders and beds in hospitals, i.e., data (confirmed and official), institutional advices and solicitation to respect the rules.

**Figure 2:**
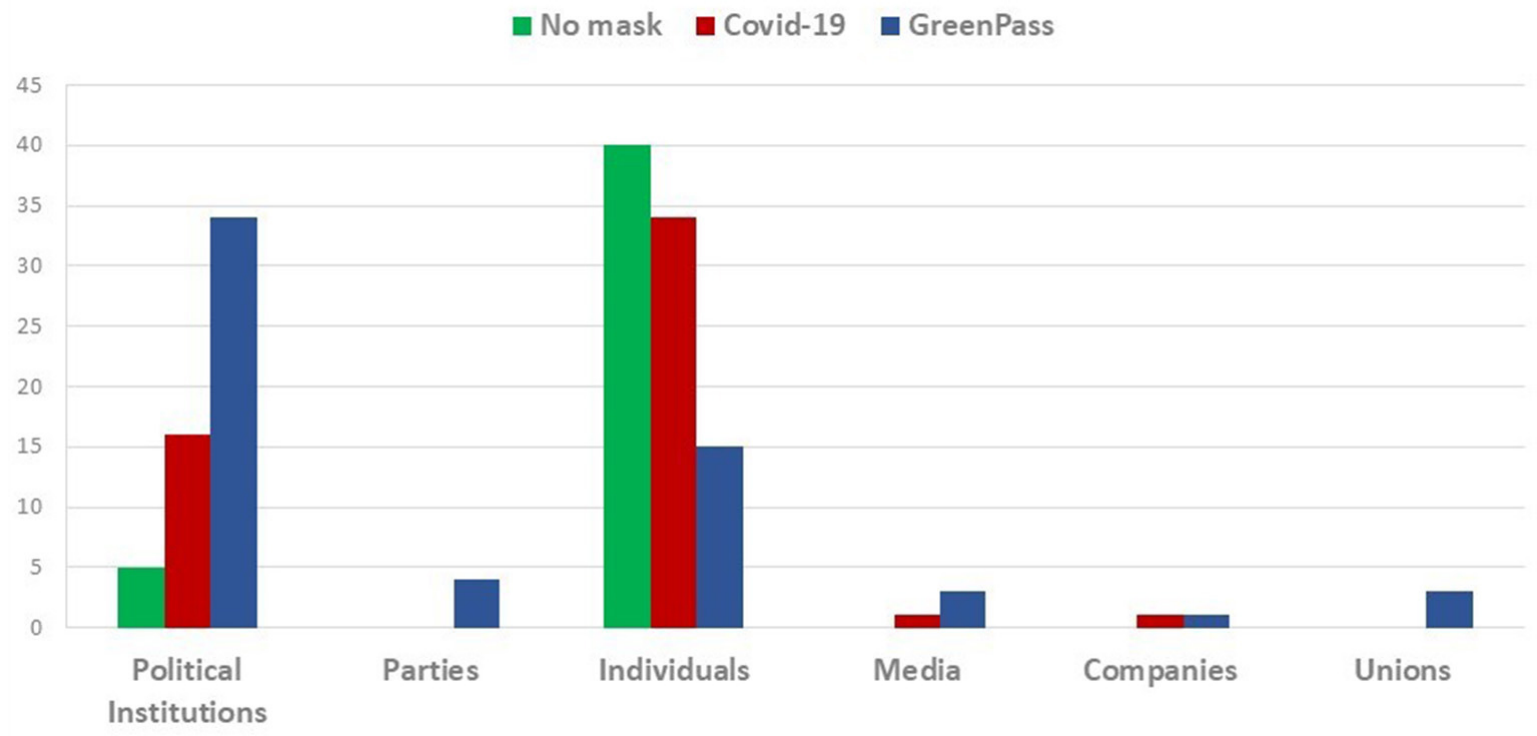
Top 10 tweets: distribution of hashtag for different recipients.

In the opposite direction, the third wave communication (focused on hashtag “Greenpass”), although coming mainly from private citizens, is evidently made up of subjects with a high sense of political effectiveness and self-directed, largely oriented to reach and “influence” political institutions and decision-makers (Figure 2).

As anticipated at the beginning of the paragraph, this first descriptive step of the analysis aimed to identify (when possible) information on geographical origins, on the sources of comments (politicians, newspapers, private citizens, etc.) and on the feelings that can be deduced from the type of users involved, main topics and recipients. Overall, it turns out that the main hubs are different for each selected hashtag.

**Figure 3:**
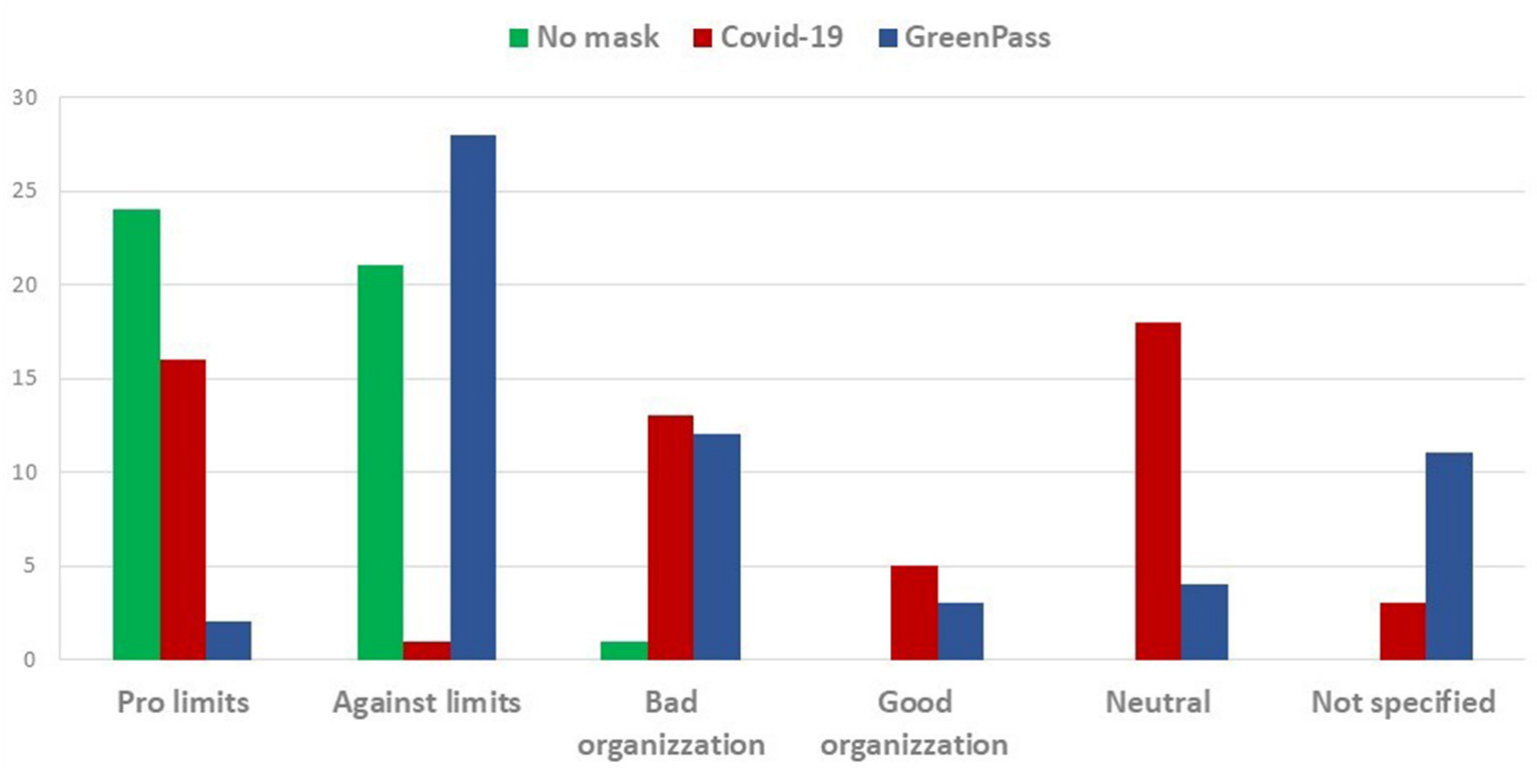
Top 10 tweets: distribution of hashtag for policy evaluations against the spread of infections.

The tweets about the hashtag “No-mask” are almost exclusively managed by private individuals and constitute networks that are not very dynamic, closed, mostly focused on small groups, with forms of communication very similar to those typically oriented by popular rumors. These networks can be further divided into two clear exclusive categories: the pro-restrictive measures that use the hashtag for derisive or ironic purposes (mostly in Italian), and the actual “No-masks,” which are opposed to any form of freedom limitation (Figure 3).

The patterns of the communication change when the “COVID-19” tweets are extracted. In this wave, neutral positions prevail and frequent is the reference to other themes other than the contagion containment policies. The comments related to “COVID-19” are more oriented to the claim of mismanagement or, again, to support the restriction measures. The most noteworthy characteristic of this network, however, remains the fact that tweets are not only posted by private users but also by institutional actors.

Finally, the communication related to hashtag “Greenpass” shows many typical traits in common with “No-mask” communication. It's mainly politically oriented movements that—unlike the latter- are localized in Italy and (at a lesser extend) in United Kingdom. Therefore, it seems to emerge a new phase with a participatory structure oriented to protest and raising from below but more polarized than the previous one.

### 3.2. The structure of communication

The choice of hashtags shed light on particular types of users, target and communication aims (information, protest, advices, etc.). At the same time, it also affects the structure of communication. For example, it is possible to identify the presence of thematic groups or real communities.

**Figure 4:**
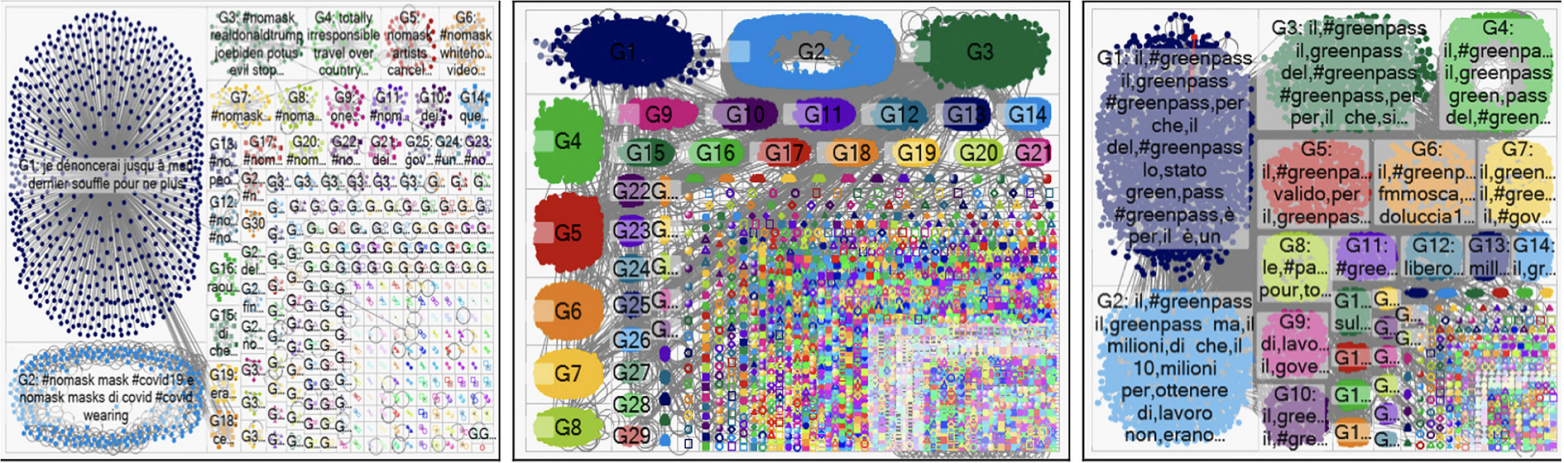
The representative graphs about “No-mask,” “COVID-19,” and “Greenpass.”

Figure 4 shows the graphs of communication network for each hashtag. The first to the left is the one related to the keyword “No-mask.” It is possible to notice how communication is divided into two parallel communities with only two main components and a series of unconnected nodes. By contrast, the communication centered on the lemma “COVID-19” (at the center) appears divided into a large number of subgroups. Finally, the “Greenpass” communication (to the right) seems to be in an intermediate position between the two with a high number of dense groups. The hypothesis is that the more divisive a topic is, the more likely it is that large groups with conflicting opinions will emerge, the more general a topic is, the less likely it is to observe polarized subgroups.

**Figure 5:**
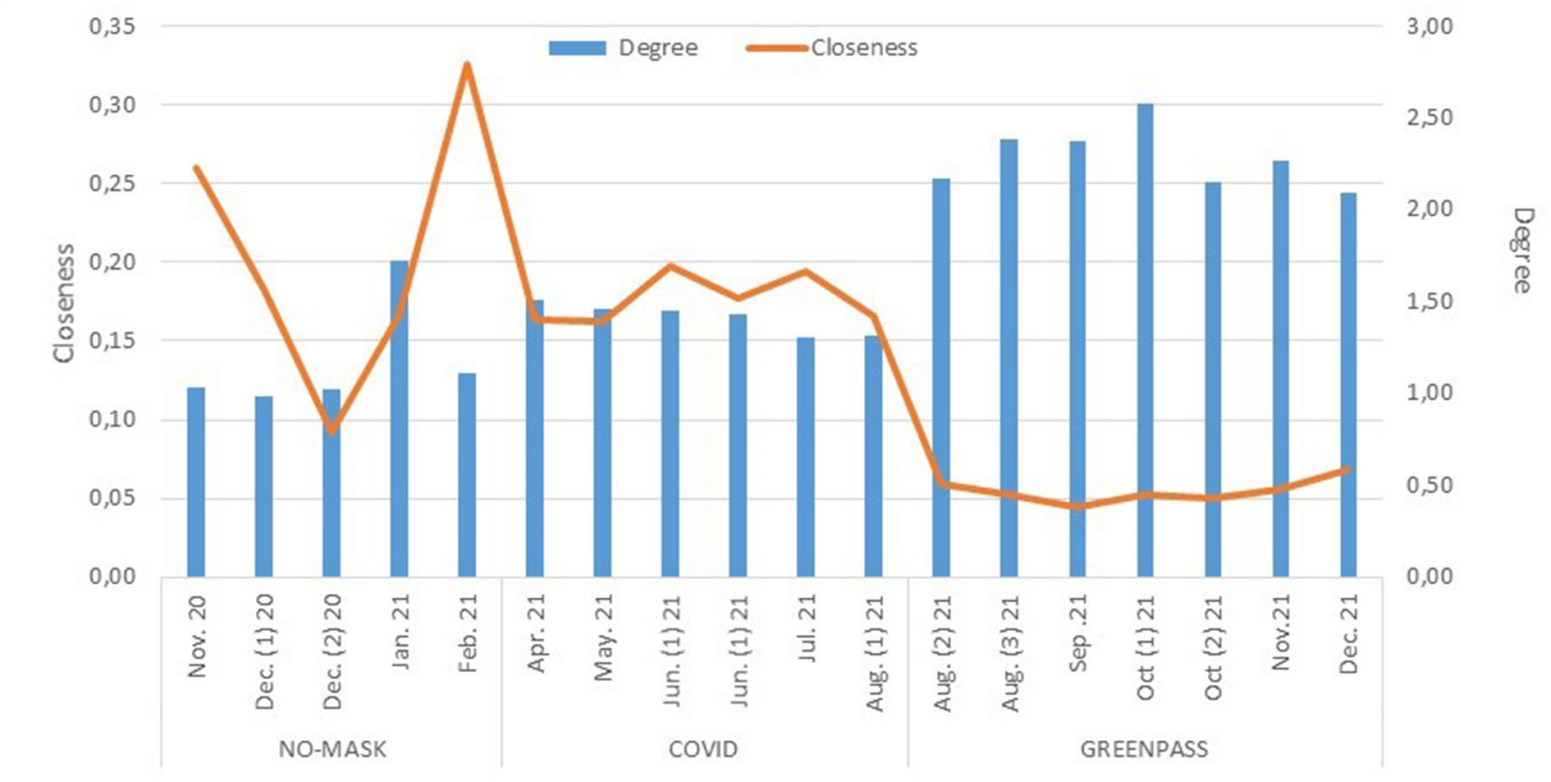
Distribution of *degree* and *closeness*. Time series: November 2022–December 2121.

To better understand the diachronic dynamics underlying the structure of the groups and their communication, the major network measurements were applied to the tweet analysis (Priyanta and Prayana Trisna, 2019). The first of these measurements is *closeness centrality*—calculated as the sum of reciprocals of the smallest distance between each node, in formula:


(1) $$\begin{array}{l} C_{c}\left(n_{i}\right)={\left[\sum_{j=1}^{g}d\left(n_{i},n_{j}\right)\right]}^{-1} \end{array}$$

where *d(n*_*i*_*, n*_*j*_*)* is the distance among the *i-*th node and all the other *g-th* nodes. So, the *closeness* provides information on the presence of thematic groups. The higher the *closeness*, the higher the presence of compact communities sharing a specific topic. On the other hand, low levels of *closeness* suggest a communication with no specific focus and small groups, which implies that most of the network's nodes rarely interact. Despite the *closeness* never reaches high values during the observed periods (Figure 5), it is true that the highest values are recorded in the period in which the hashtag “No-mask” was observed. This would seem to be in line with what the graphs suggest (Figure 4).

During the “No-mask” detection period, in fact, few distinct thematically polarized groups (but with many nodes) are observed. By changing hashtags, inserting a more general topic (such as COVID-19) communication is “pulverized” into as many subgroups as possible subtopics. A whole different speech deserves the “Greenpass” hashtag. As said, it mainly concerns Italy, it is addressed to institutional actors (Figure 2) and it was carried on at that time when the phenomenon was considered outdated. It may not be a coincidence that in November and December (restarting the infections) there is a reversal of the *closeness* trend as consequence of debate resumption.

The second network measure employed in the analysis is centrality *degree*, here used in standardized form. Centrality *degree* is obtained considering the number of linkages each node has—d(*n*_*i*_) on the total amount of possible linkages underneath the network (g-1), in formula:


(2) $$\begin{array}{l} C_{d}^{{}^{\prime }}=\frac{d\left(n_{i}\right)}{g-1} \end{array}$$

In the specific case, centrality *degree* refers to the number of relations among nodes, detected looking at the reactions to the tweet, i.e., visualization, retweet, likes, etc. (Bild et al., 2015). As it is possible to notice from Figure 5, the *degree* shows a trend growing over time. Such an increase seems to be triggered by the specific topics observed and it reaches its acme when dealing with communication on “Greenpass” (Figure 5).

The “No-mask” communication does not appear centered on few particular nodes but, as shown by the *closeness*, it will generate few discussion groups containing very homogeneous nodes in thematic terms. The low *degree* values—in fact—suggest a peer communication within groups and not centered on public users. Moving on to analyse the *degree* in “COVID-19” communication, there is a slight value increase compared to the previous period. This could be due to the “source” nodes of this communication. As observed in Figure 1, in this period the debate is often generated by political and institutional actors whose visibility consequently triggers a greater chance of reaction, both in terms of protest and in terms of inspiring the wish to go deeper (Miller, 2020).

The *degree* centrality reaches its peak in October (2, 57) when observing the network structures related to the third focus of the analysis: the hashtag “Greenpass.” The high *degree* value, in this case, highlights the existence of a broad and open communicative dynamic. In such a case, communication does not only imply reactions—which are still present—but it becomes more direct, creating an actual debate between private and public users which communicate with one another in the “digital square” and not in close subgroup as observed in the “No-mask” network.

**Figure 6:**
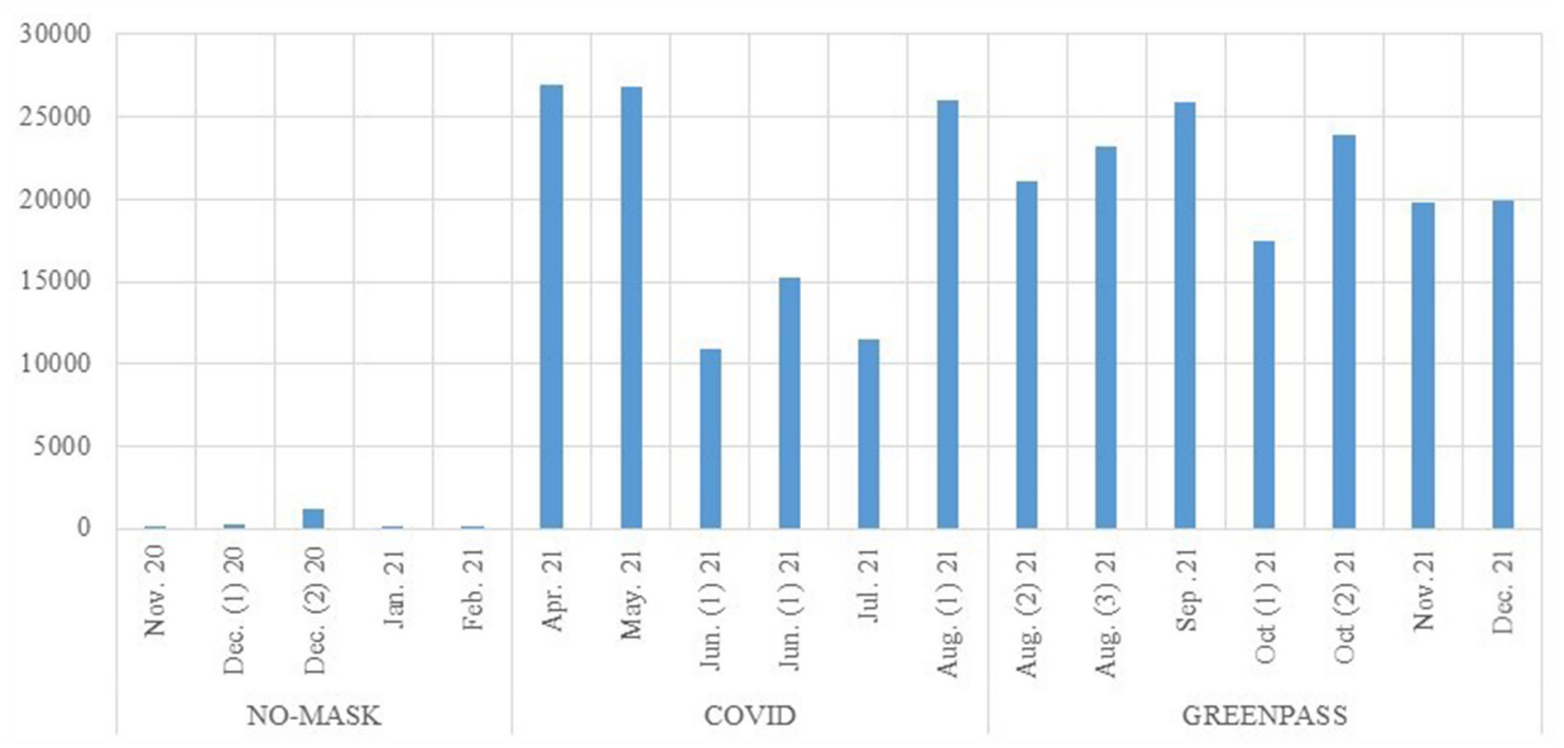
Distribution of *betweenness*. Time series: November 2022–December 2121.

The third measure used in the analysis is the *betweenness* centrality. It is obtained through the sum of all of the partial *betweenness* calculated for each couple of nodes, in formula:


(3) $$\begin{array}{l} C_{b}\left(n_{i}\right)=\sum_{j<k}g_{jk}\left(n_{i}\right)/g_{jk} \end{array}$$

where *g*_*jk*_(*n*_*i*_) is the number of geodesics that connect two nodes containing a *i-*th node. *Betweenness* centrality highlights the presence of users that play an intermediate role between either users or groups of users. A high value of *betweenness* indicates an open communication network characterized by continuous exchanges between nodes and between subgroups. On the other hand, a low value of *betweenness* underlines the presence of closed groups not interested in communicative exchange with other groups. The analysis of *betweenness* in “No-mask” period (Figure 6) confirms what has been previously noted. In this case, in fact, the communicative structure seems sparser, a sort of pseudo-dialogue among people sharing the same thoughts. Closed groups within which no nodes with intermediary roles are observed and reluctant to accept exchanges with other groups.

On the other hand, communication in the following two periods appears more intense. The presence of intermediaries emerges, in fact, when looking at the “COVID-19” centered communications and becomes evident with “Greenpass” centered discussions. The involved nodes, in this case, not only participate by reacting to tweets but sharing them contributes to their dissemination and in some cases generates protest movements on the digital and real streets.

### 3.3. The main contents

**Figure 7:**
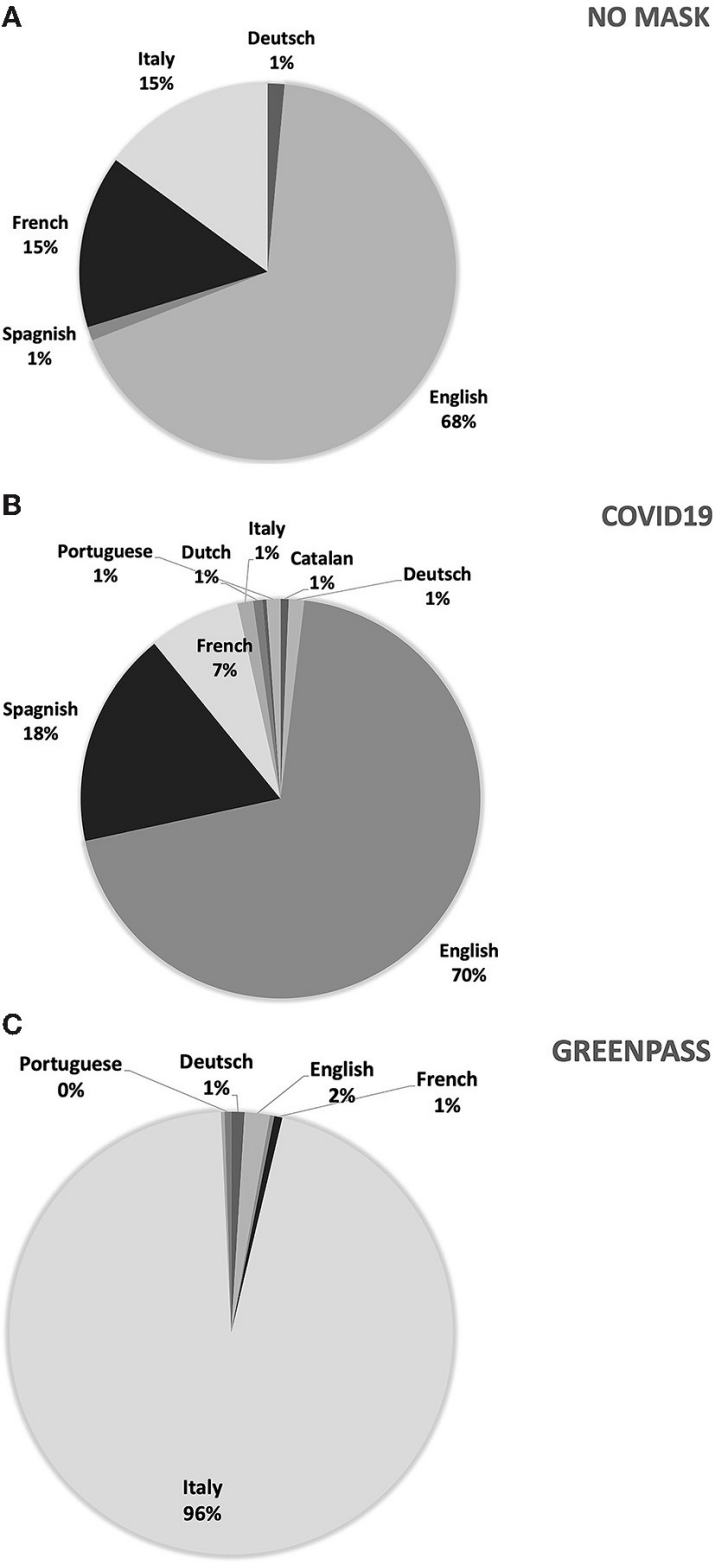
**(A–C)** Comments for languages (% on European languages).

In order to analyse the content of the comments disseminated in the networks, we opted—finally—to conduct an analysis on the main languages, for each of the selected lemmas. The structure of the communication is different, depending on the lemma identified, and the contents are not necessarily aligned. Highlighted dynamics and emerged points of view are very different, depending on the reference context. First of all, the prevailing languages are not always the same. If we restrict the analysis to European languages, it emerges that most of the “No-mask” comments are in English, Italian and French and those on “COVID-19” are mainly in English and, to a lesser extent, Spanish. Those centered on the word “Greenpass” are, however, predominantly in Italian (Figure 7).

The first analyzed lemma, “No-mask,” mainly involves (reciprocal) communication between private users. That said, the structure and content of communication are identified progressively, moving from the analysis of associations between basic units of communication (co-occurrences between lemmas), to the identification of clusters of main phrases (elementary contents of communication).

The analysis of co-occurrences centered on the abstraction key allows us to detect some content specificities. The meanings related to the keyword “No-mask,” for example, change depending on the languages. In Italian there is a clear association with no-vax, conspiracy, but also with a series of terms used to ironize against the self-styled No-mask movement (manginobrioches and terrapiattisti).

The French communication is deeply different: the ironic element disappears and it is replaced by the emotive one, particularly with respect to the limitations imposed on children (enfant, coronaprincess, and denoncer) and the reference to historical events traceable to wars and revolts (resistance and genocide). The meaning of these associations is not always evident, but becomes clear in the other phases of the analysis when phrases and comments emerge, which are also traced back to precise links and references.

Further specificities are noted with the analysis of the English comments, where the content mainly concerns the dissent for the restrictions on freedom of movement in front of the social and relational events in which one would like to participate. The liberal matrix of dissent is evident, with protests about shop closures or against the ban on entry without a mask, but also references to the assumed uselessness of these decisions (*#maskdontwork*).

These differences emerge more clearly when comparing the clusters of contents for each context (i.e., language). This is possible thanks to the Multiple Correspondence Analysis (MCA) which, applied to lexical content, shows the association between main lemmas and periods and also the most repeated content.

**Figure 8:**
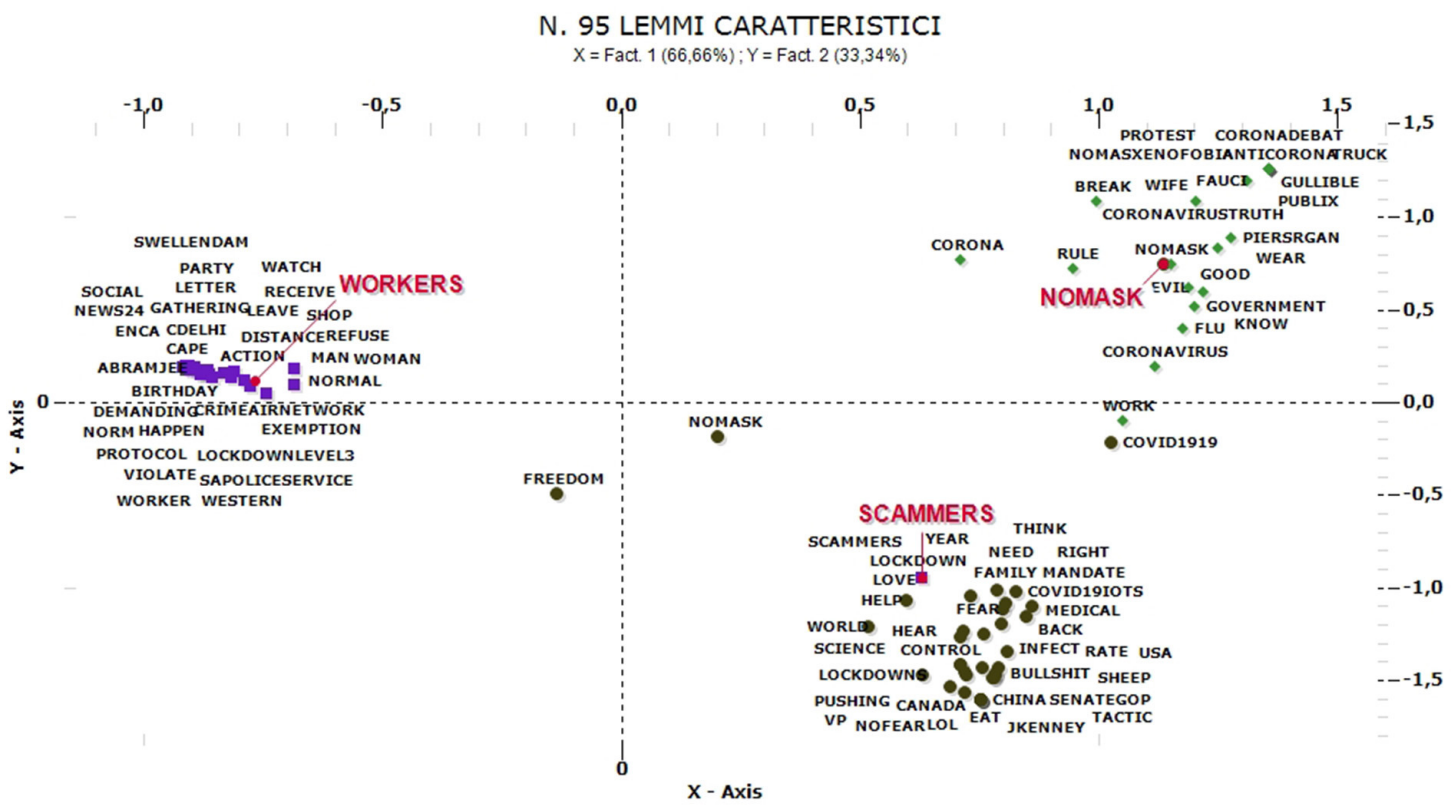
ACM—“No-mask” comments in English (95 characteristic lemmas).

So we identify the main clusters for comments in each main language (for the “No-mask” comments, we defined three main linguistic structures in English, French and Italian). The most numerous comments are the English ones. The clusters obtained from the ACM are three and are named as: “No-mask,” “Scammers,” and “Workers” (Figure 8).

The “No-mask” group (26% of the comments relate to this cluster) is formed between November and December, when protests over freedom restrictions characterize communication. In this group there are controversial comments against restrictions and/or vaccines and opposes the cluster called “Workers” (the main one, 45% of the contents) referring to the protests against the limitations of work activities but also, more generally, to the closure of shops/movements. Many of these comments refer to a video showing a woman being kicked out of a shop for refusing to wear a mask. Comments related to this cluster, mostly from January, are associated with protests about rules, protocols and the impact on work/life/leisure. This cluster is particularly numerous and includes both protests against the containment measures/regulations for workers, and comments referring mainly to labor discipline, in particular subsidies and business support. Finally, the third cluster, “Scammers” (29%), consists of comments referring to fears of contagion and protests against public or private figures who violate the rules of distancing (parties, use of masks in public places, etc.).

**Figure 9:**
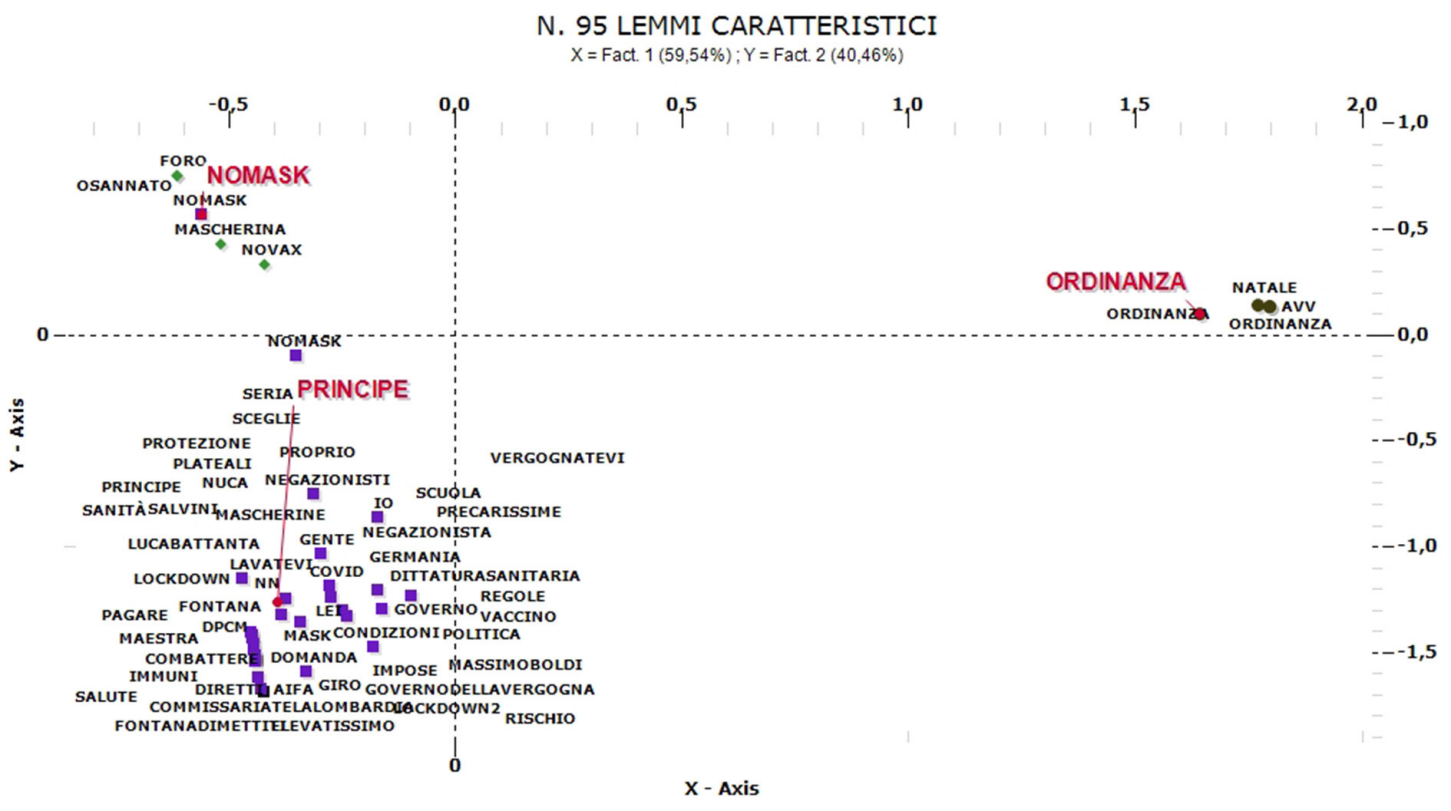
ACM—“No-mask” comments in Italian (95 characteristic lemmas).

Looking at Italian comments (Figure 9), the cluster named “No-mask” (19% of contents are related to this cluster) concerns mainly comments of January and February. Here appear polemic messages against the restrictions and the use of DPI, news and statistics supporting No-mask thesis. Only this little group actually support No-mask movement. The other two clusters are against No-mask movement and/or no-vax positions: “Principe” (57%)—with ironical languages and political references (the most part, against No-mask topics)—and “Ordinanza” (23%), referred to political scandals, the misuse of public funds or real errors of assessment that caused increases in infections and number of deaths among frail elderly people. The ironic component is the most frequent, both against No-mask movement (mainly) and political personalities.

**Figure 10:**
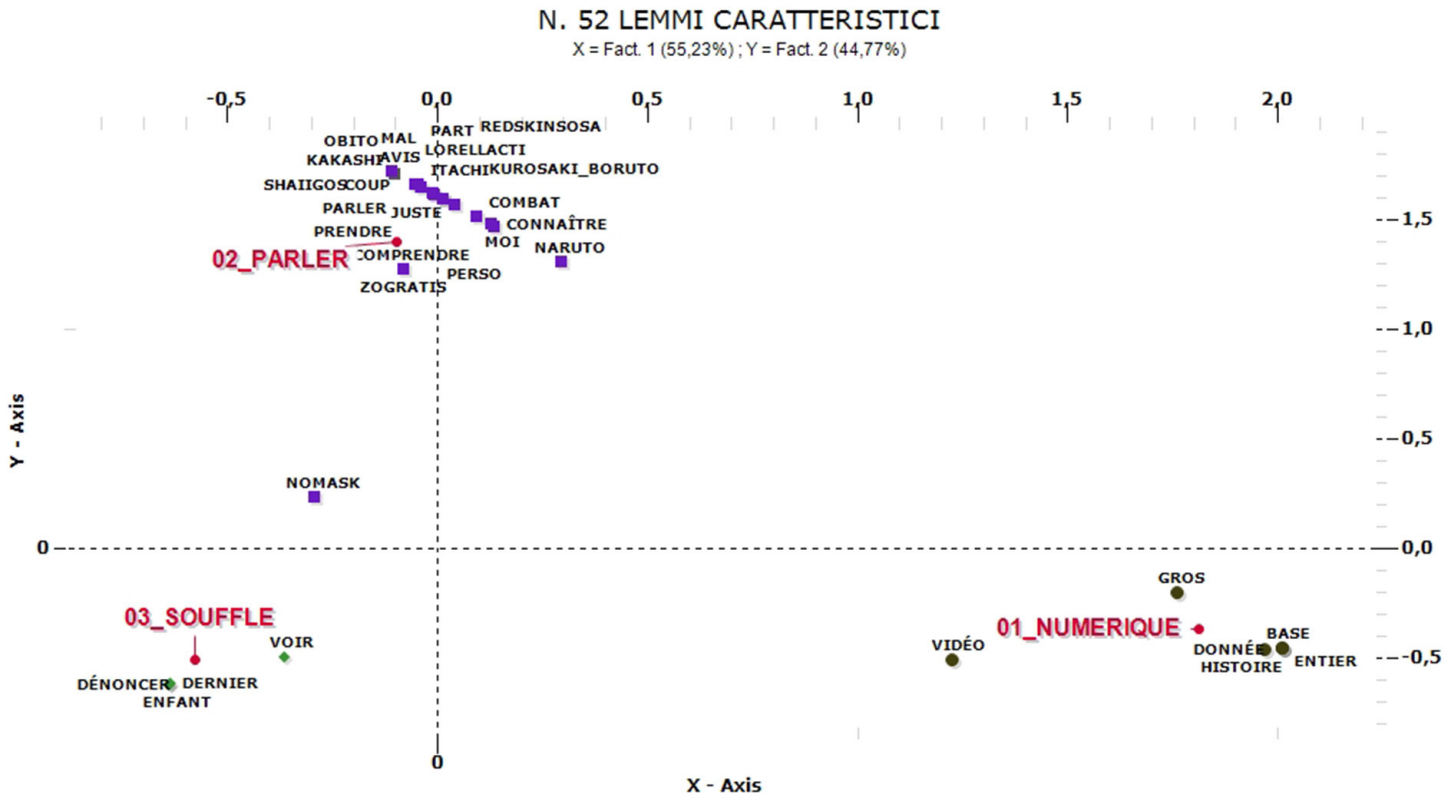
ACM—“No-mask” comments in French (52 characteristic lemmas).

Instead, the comments in French shows a huge weight of emotional states. The analysis shows three clusters of comments (Figure 10) but the main thematic group, named “Souffle” (52% of contents are related to this cluster, mainly posted in December) is the most emotional one: it is referred to limitations and distances imposed in schools and among children even in kindergartens (*enfant* and *dénoncer*). The other relevant cluster is also the less numerous cluster is named “Numerique” (17%) and it refers mainly to fake news on data about contagion and conspiracy hypotheses, with inappropriate references to historical events as Nazism and related limitations of quite different meaning and scope (similar considerations also emerge in Italy but in relation to the keyword “Greenpass”). A further cluster named “Parler” (31%) occurs mainly in February and it is not referred to pandemic crisis at all. It includes mainly comments, discussions and debates among young people referred to pop culture and Japanese culture.

Considering the second phase of the research—centered on the word “COVID-19,” it has already been noted that these comments are very numerous and heterogeneous. Some of them (quite marginal) also refer to protests against distancing measures. These comments are, however, mostly in support of decisions aimed at reducing contagion. Restricting to European languages, the most numerous Tweets are, predictably, those in English. They are followed, however, by those in Spanish. Focusing on these two idioms, typical contents of communicative clusters can be identified.

First of all, analyzing the words in English, the reference to health fears certainly emerges (patient, pandemic, health, case, death, help, and hospital). This is the prevailing content with respect to the proposed extraction, juxtaposed with the different positions for and against vaccination and containment measures, a reference strongly associated with the Indian context, which suffered many losses and had particular difficulties in managing the emergency (support, vaccination, and mask). The reference to India, unlike other areas with similar problems, is very much present on social media and as comments in English, aimed to promote activities for the population. A third area is about information and reference to statehood (report, and State).

Reference to vaccination and health protection concerns are also widely present in the comments in Spanish, which, however, refer to the local (*pais*) rather than the national dimension and to the level of reflection and respect for the person (*Dialogoporlapaz, persona*, and *medidas*).

**Figure 11:**
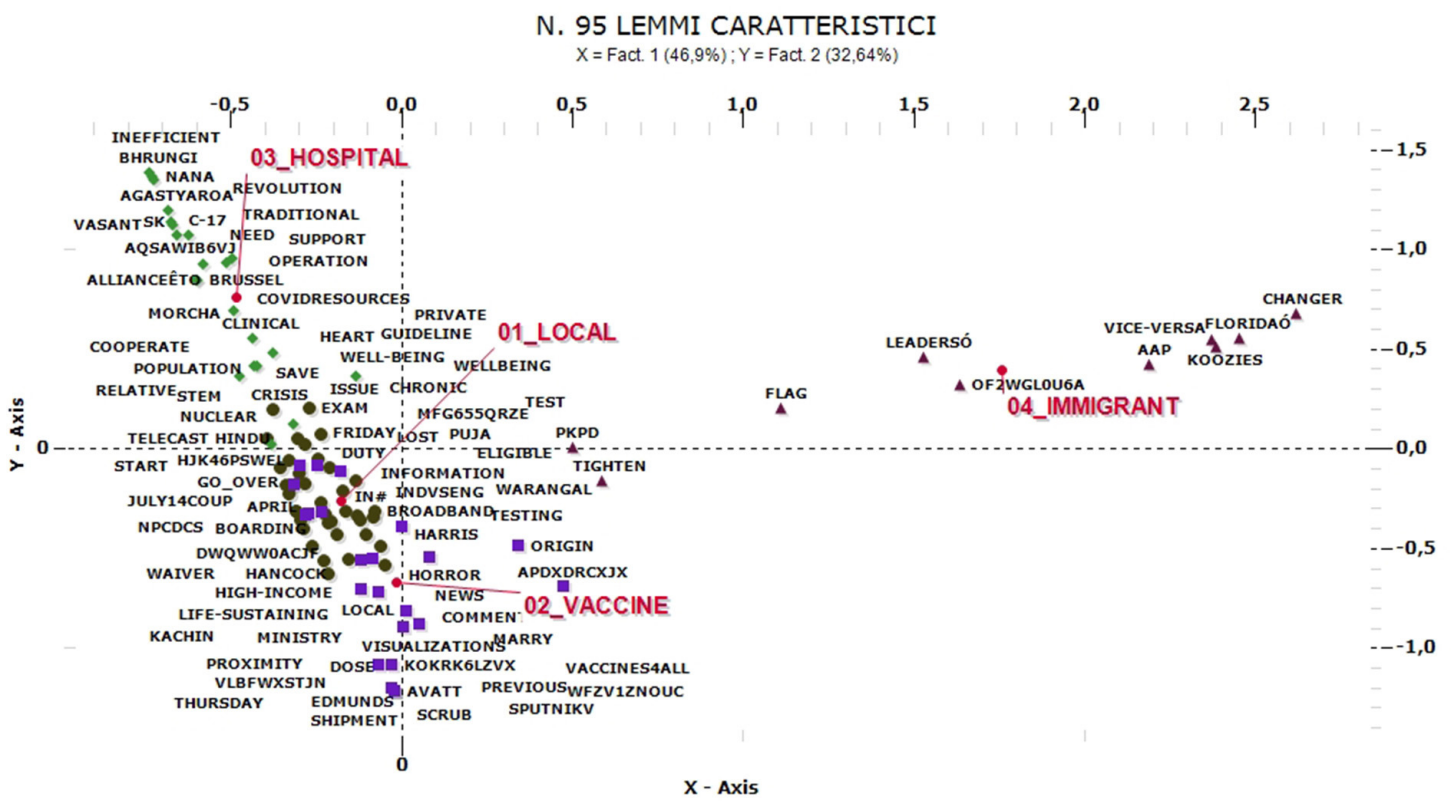
ACM—“COVID-19” comments in English (95 characteristic lemmas).

Further information can emerge from the A on elementary contexts, through which it is possible to distinguish four thematic clusters for each idiom (Figure 11). In English, the “local” cluster prevails (32%), which refers to local statistics on infection rates distributed throughout the territory, reports and similar references and which is characteristic of May and July. This is followed by the clusters “hospital” (26%—April) and “vaccine” (24%—August), which are of obvious interpretation, and the cluster “immigrants,” which refers to planned indications for the treatment of sick immigrants or limits on access, quarantines or, again, to the risk that the presence of irregular immigrants causes an increase in contagions. These comments vary widely in relation to the reference context, from Texas to Canada. The latter cluster is specific and isolated from the others and it is not present in any other analysis but only emerges—with this centrality—in relation to the English-speaking context.

**Figure 12:**
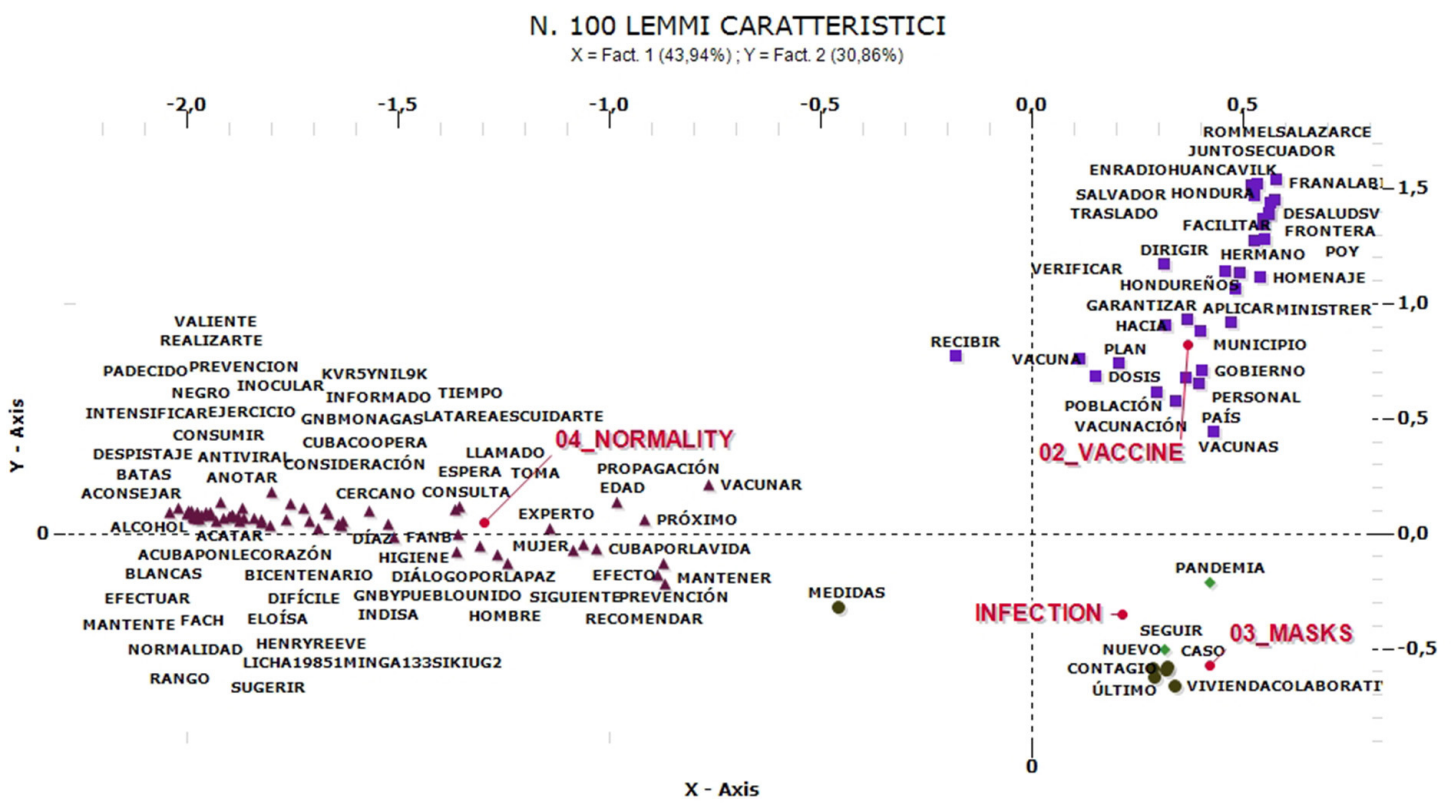
“COVID-19” comments in Spanish—Multiple Correspondence Analysis.

Looking at the co-occurrences, the comments in Spanish would rather seem to be mainly centered on concerns about contagion and on the issue of personal protection. These comments are clustered in four groups (Figure 12) called “infection” (33%), “vaccine” (22%), “masks” (19%), and “normality” (26%). The prevailing themes are thus clearly aligned with concerns about infection and the desire to resume a life considered normal. This is followed by heterogeneous reflections on the vaccine and there are not significant clusters with protests toward regulations and controls.

Finally, the last stage of monitoring concerns the “Greenpass” extraction key, which is associated both with issues specifically linked to the Italian polemical debate against restrictions (no-Greenpass, and no-vax), institutional references (government and Italy) and the desire of protect freedom (obligation, freedom, and impose). These elements also emerge, all of them, from the Multiple Correspondence Analysis. This second procedure shows the multi-semantic nature of the movement against compulsory certification, which is particularly stringent in the Italian context.

**Figure 13:**
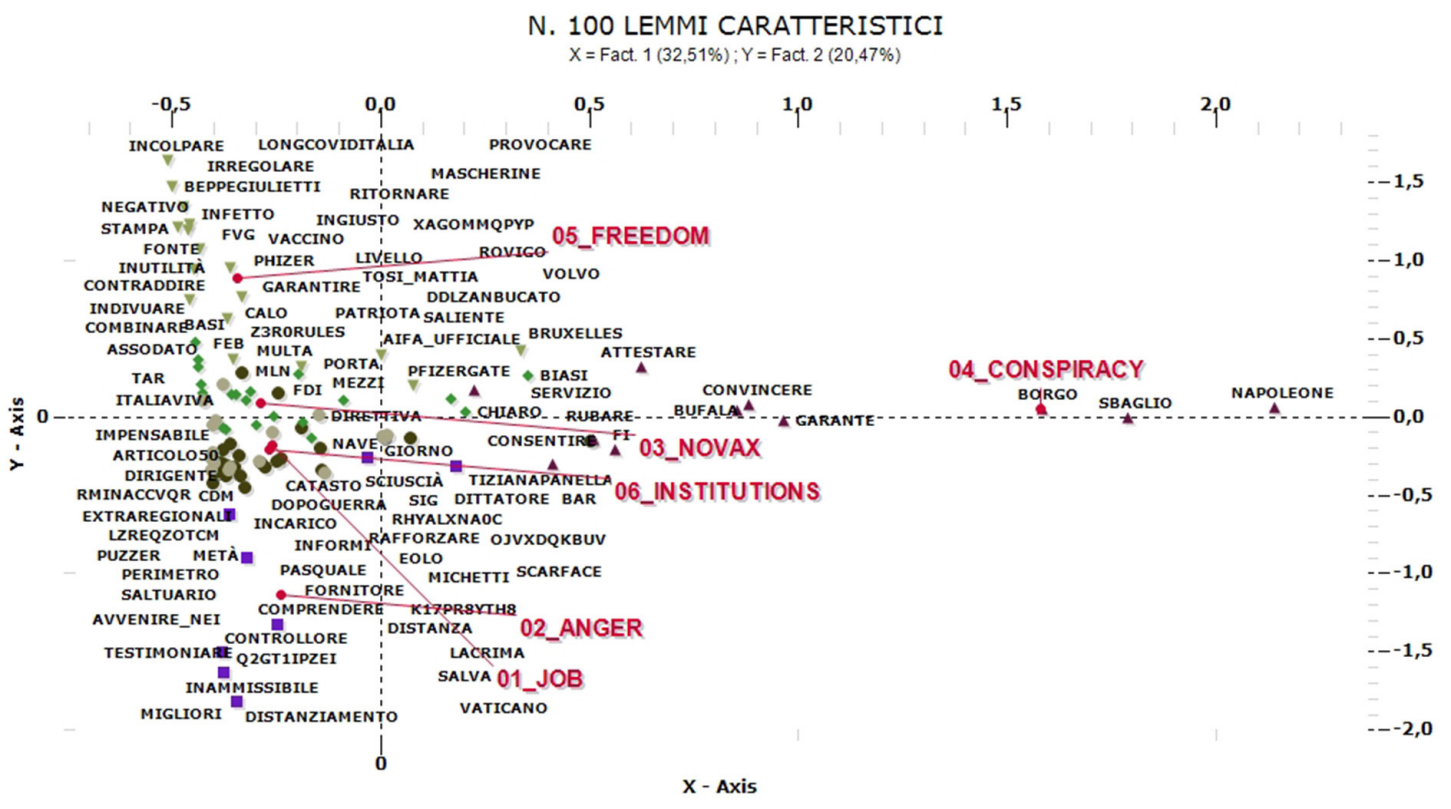
ACM—“Greenpass” comments in Italian (100 characteristic lemmas).

In fact, six thematic clusters can be distinguished (Figure 13). The cluster named “job” (16% of comments) includes tweets that deal with practical issues such as access to workplaces with a certificate or the rights of the worker after the application of the COVID-pass obligation, while the cluster named “anger” (12%) includes comments that complain about constraints and obligations using particularly aggressive language and proposing inappropriate parallels, such as references to limitations of freedoms typical of dictatorial regimes or historically known references to war events and forms of xenophobia or genocide (the same structure of communication emerged for some French tweets belonging to the “numerique” cluster in the “No-mask” communication).

The “no-vax” cluster (16%), on the other hand, refers above all to expressions of protest by those who do not want to be vaccinated. Another cluster identified can be considered related with this one and in named “conspiracy” (25%). This includes comments referring to alleged conspiracies aimed at imposing constraints and limitations for reasons completely unrelated to health risks, seen as a non-existent or overstated threat in order to impose behaviors, political positions and uncomfortable decisions, even of economic nature (sometimes explicitly recalling the theme of health dictatorship). Similar arguments but mainly centered on the protection of rights and freedoms characterize the fifth cluster, named “freedom” (18%), which refers to topics particularly mentioned in August, while the last cluster includes institutional references (12%), with references to politicians, government and healthcare, both in polemical and neutral terms.

Overall, communication is mainly centered on forms of protest and social and political disaffection. Clusters referring to the need to work, the risk of economic crisis and political recriminations (“job,” “anger,” and “institutions”) prevail in October, while those referring to limitations as an unnecessary or unjustified constraint are present in summer (“freedom” and “no-vax” in August, “conspiracy” in September). It is interesting to note how this plethora of protests (both rational and irrational) and even instrumental motivations are characteristic of a context that, at the beginning of the extraction, was rather critical of the No-mask movement itself (now represented in the “conspiracy” and “no-vax” clusters). These are, therefore, two very different phases that drive positions and opinions decidedly far apart. Clusters with a strong polemical and emotional content are also the most numerous, followed by those referring to economic and political motivations (“job” and “freedom”).

## 4. Discussion

The proposed work reconstructs the debate developed on the social network twitter and related to issues associated with the pandemic crisis. Several studies have, recently, focused on both reconstructing narratives and analyzing attitudes and behaviors arising from the particular emergency conditions (Larson, 2018; Ball, 2020; Velotti et al., 2022). In addition, many works, beyond the emergency plan and specific social, political, and economic reactions, have dealt with the pros and cons associated with the spread of communication on social networks, particularly with reference to the dissemination of distorted information (Kata, 2010; Chou et al., 2018; Al-Ramahi et al., 2021; Herrera-Peco et al., 2021) and, more specifically, with regard to the risks of cognitive fallacy associated with a confirmation bias (Bessi and Quattrociocchi, 2015; Del Vicario et al., 2016).

The paper traces the main theses and considerations that have emerged in reference to the widespread perception of the pandemic crisis and different modes of reaction, identifying three specific, interdependent topics: the perception of risk and the construction of scapegoats; reactions typically related to ontological insecurity (from reference to ideologies to the spread of fake news); and the emergence of reinforcing effects of fallacious reasoning dependent on unconscious overexposure to customized and catchy messages. The latter dynamic, in particular, is studied with reference to the algorithmic management of communication on social media.

Having posed these arguments, the study seeks to reconstruct the main lines of argument of communication on social, also identifying them with respect to the characterizing periods. To this end, rather than automatically referring to the over-exposed arguments on social, it was decided to identify the main extraction keys as a result of continuous monitoring of media communication but also of communication on social. A second reason for the selection derives from the attempt to identify different forms of communication, distinguishing—specifically—three of them:

- #No-mask: a strongly distorting communication with respect to the content of institutional and health information in particular, characterizing a transnational collective movement and protest, as ephemeral as it is lively in its expansion phase but destined to be short-lived (after a few months there is an evident reduction of the reference network, at least on twitter). Comments on the social, moreover, present an ambiguous content with positions that are sometimes oriented to ridicule or irony rather than to support the ideas of reference (this is particularly the case in Italy, an area particularly affected, in the first phase, by mortality and contagion);
- #COVID-19: this second communicative form characterized the entire emergency period and still remains significant on the web; it mainly consists of comments supporting institutional and prudent messages or the institutional messages themselves, oriented to inform and spread prudent practices;
- #Greenpass: a third communication structure, is specifically prevalent in the Italian context, where control measures have been particularly incisive and long-lasting, even after the distribution of vaccines and therapies. Particularly at a time when there appears to be too much caution compared to other areas (particularly liberals) that have limited or eliminated forms of distancing and progressive closures, communication on the issue becomes particularly tight and also produces off-line forms of protest, constituting real communities of discussion if not an actual protest movement.

The three conditions of online communication reflect, in effect, three phases of action and reaction to the pandemic threat: alarm, emergency and generalized restriction of activities; protection of the population and institutionalization of the emergency; and progressive return to normality and openness (which entails increasing criticism where a condition deemed not justifiable prudence persists, even in the face of news contexts where constraints are already removed, with serious socio-economic disruptions at the local and national levels).

Finally, on the methodological level, it is possible to identify some specific advantages of the three-step procedure proposed here, which allows for the identification of messages and communications constitutive of comparison networks, minimizing the “noise” that is always present when extracting information on big data.

The analysis is circular and open. Each step confirms elements that emerge from the others so you can modify the parameters when changes in the style and structure of communication occur. Specifically:

- the extraction of the Top-10-tweets allows an in-depth analysis of a low number of relevant comments. So we quickly identify errors, problems and new topics (in February 2021 #No-mask became almost irrelevant as twitter reference);
- the network analysis tools allows us to identify the structure of communities and how they change over time, selecting the most important communities or discussions, the differences among structures of information, self-reference and communications, the presence of hubs within the net, etc.;
- the automatic content analysis on specific languages allows us to evaluate with attention the content of comments and the importance of each cluster (also in terms of debate and construction of parallel communities), comparing communication through main topics, languages and periods.

Finally, we obtain the main information related with the structure of communications, the main languages, the main problems related to information and—where appropriate—the symptoms of institutional disaffection or actual social protest. It is therefore possible, using this procedure, to identify information, indicators and narratives referable to political and social debates involving different categories of users, identifying the main sources (institutional and non-institutional, if private profiling with respect to useful information), motives, suggestions. This practice could also be useful in acquiring information for implementing social policies or public decisions, identifying problems or reshaping structure and content of messages and information for the population.

## Data availability statement

The raw data supporting the conclusions of this article will be made available by the authors, without undue reservation.

## Author contributions

RD'A is responsible for the Sections 3.1, 3.2, and 4. SG is responsible for the other parts. All authors contributed to the study design and approved the final version of the manuscript.
